# Proteomics-based discovery of biomarkers for paediatric acute lymphoblastic leukaemia: challenges and opportunities

**DOI:** 10.1111/jcmm.12319

**Published:** 2014-06-09

**Authors:** Elena López Villar, Duojiao Wu, William C Cho, Luis Madero, Xiangdong Wang

**Affiliations:** a Hospital Universitario Infantil Niño JesúsMadrid, Spain; b Biomedical Research Centre, Fudan University Zhongshan HospitalShanghai, China; c Department of Clinical Oncology, Queen Elizabeth HospitalKowloon, Hong Kong; d Department of Respiratory Medicine, Zhongshan Hospital Fudan University School of Medicine, Shanghai Respiratory Research InstituteShanghai, China

**Keywords:** acute lymphoblastic leukaemia, proteomics, biomarkers, paediatrics

## Abstract

There are important breakthroughs in the treatment of paediatric acute lymphoblastic leukaemia (ALL) since 1950, by which the prognosis of the child majority suffered from ALL has been improved. However, there are urgent needs to have disease-specific biomarkers to monitor the therapeutic efficacy and predict the patient prognosis. The present study overviewed proteomics-based research on paediatric ALL to discuss important advances to combat cancer cells and search novel and real protein biomarkers of resistance or sensitivity to drugs which target the signalling networks. We highlighted the importance and significance of a proper phospho-quantitative design and strategy for paediatric ALL between relapse and remission, when human body fluids from cerebrospinal, peripheral blood, or bone-marrow were applied. The present article also assessed the schedule for the analysis of body fluids from patients at different states, importance of proteomics-based tools to discover ALL-specific and sensitive biomarkers, to stimulate paediatric ALL research *via* proteomics to ‘build’ the reference map of the signalling networks from leukemic cells at relapse, and to monitor significant clinical therapies for ALL-relapse.

- Introduction
- Deciphering ALL pathways *via* proposed proteomic strategies
- Examples of leukaemia research by using proteomics
- Conclusions and future perspectives

## Introduction

Acute lymphoblastic leukaemia (ALL) is the most common malignant disease diagnosed in children and represents one-third of paediatric malignancies. There are still around 30% of the patients to be relapsed, even though therapies for leukaemia have been improved over last decades. Twenty per cent of relapse cases have an isolated extramedullary relapse, of which the central nervous system relapse is about 65% [1]. Three main prognostic factors are considered in the outcome of first ALL-relapse patients, including the time of the initial diagnosis to relapse associated with better prognosis in late relapses, the location of the relapse with better prognosis for extramedullary relapses, and the immunophenotype of the leukemic cells with worse prognosis for T-cell phenotype. Treatment of relapse dependent on those prognostic factors includes chemotherapy and bone marrow transplant in patients with high risk of early and late relapses with poor chemotherapy responses. The characterization of the relapse leukemic blasts at an extramedullary site has been defined *via* the polymerase chain reaction (PCR) of markers, *e.g*. immunoglobulins and T-cell receptor gene rearrangements.

PCR-based analysis of minimal residual disease (MRD) is used to detect residual leukemic cells can be detected by during therapy or even single leukemic cell. Emerging technologies have been developed rapidly to enable to detect circulating tumour cells in various cancers [2]. It is essential to accurately predict patients to differentiate risk groups to optimize the strategy, as paediatric ALL is a heterogeneous disease with varied response to treatment. Risk stratification is classified into the standard, low, intermediate, or high, based on molecular and/or cytogenetic markers (*e.g. BCR-ABL* and *MLL-AF4* rearrangements) and responses to treatment. Chromosomal irregularities are frequently involved in non-random chromosomal translocations to produce new gene fusions or cause inappropriate expressions of oncogenes or altered correspondent proteins. Genetic alterations [*e.g*. t(9;22), t(1;19), t(12;21) and the rearrangement of the MLL gene on chromosome 11q23] have been suggested to impact the prognosis of patients [3–6].

Risk-based therapy is emphasized in therapeutic protocols for paediatric ALL to reduce the toxicity in patients with low risk and provide aggressive therapies for those with high risk. Age, initial white blood cells, ALL subtype, chromosomal aberrations, or MRD have been considered in the risk stratification, although the exact disease-specific and sensitive biomarkers remain unknown [3–11]. Proteomics is an opening and new window to make the discovery and identification of protein-based biomarkers possible in paediatric ALL-relapses, and a useful tool to develop individual and personalized therapies [12]. Enlightenment and complete comprehension of cell-signalling pathways and activation/deactivation are the key for discerning the progression, remission, or relapse of ALL, since cell-signalling pathways regulate and control cell proliferation, differentiation, survival and apoptosis [13].

Signalling pathways are controlled by post-translational modifications (PTMs) *via* phosphorylation of protein kinases and phosphatases. Functional pathway-mapping methodologies allow direct measurements of the activation/deactivation of proteins in signalling transduction pathways, with a great promise for discovery and identification of altered signalling pathways in ALL cells after the occurrence of relapse. Proteomics can be used to search new therapeutic targets for drug discovery and development and identify ALL-relapse-specific biomarkers earlier, and develop specific inhibitors for targeted signalling in patients with relapse.

Protein activation/deactivation is hardly analysed directly through gene-expression profiling, since PTMs are not predictable from gene expression [14]. Strategies of phosphoproteomics can be used to profile the activation/deactivation of key molecules in signalling pathways of leukemic cells from ALL patients between stable remission and relapse. A reference map of activated/deactivated pathways associated with clinical ALL-relapse can be created. Our proposed strategy allows to measure the phosphorylation levels of key signalling proteins and to identify mutated protein-residues at diagnosis, during chemotherapy, or at the end of chemotherapy to complete remission and/or relapse. The strategy can be performed in cerebrospinal-fluid, bone-marrow, or serum, *via* injection in the mass spectrometer. We have a correct simple sample study design of ALL-relapse for clinical proteomic research to get the ‘reference-signalling map’ of ALL between remission and relapse.

### Deciphering ALL pathways *via* proposed proteomic strategies

Several signalling pathways (*e.g*. PI3K/AKT/mTOR, JAK/STAT, ABL tyrosine kinase, SRC family of tyrosine kinases or NOTCH1) regulate and control the activation, proliferation and survival of B and T cells during ALL. Leukemogenesis is controlled *via* the regulation and interaction of those signalling cascades as a network. mTOR activity increased at ALL-relapse and was suggested as the therapeutic target to design new drugs for human solid cancers or lymphoid malignancies, including ALL. Recent evidence showed that Cyclin E up-regulated in patients in the early stage of relapse, corrected with poor prognosis [15]. The signalling pathways have been considered to be associated with ALL progression [16]. There is a limited understanding of the critical role of proteins associated with the activation of signalling pathways and with the network-based interaction in the diagnosis and prediction of ALL-relapses. Of omics tools, phosphoproteomics are applied to identify and discover protein-based biomarkers of ALL-relapse and explore functions of targeted proteins in cells, tissues, or organs to improve therapies [17–28].

**Fig. 1:**
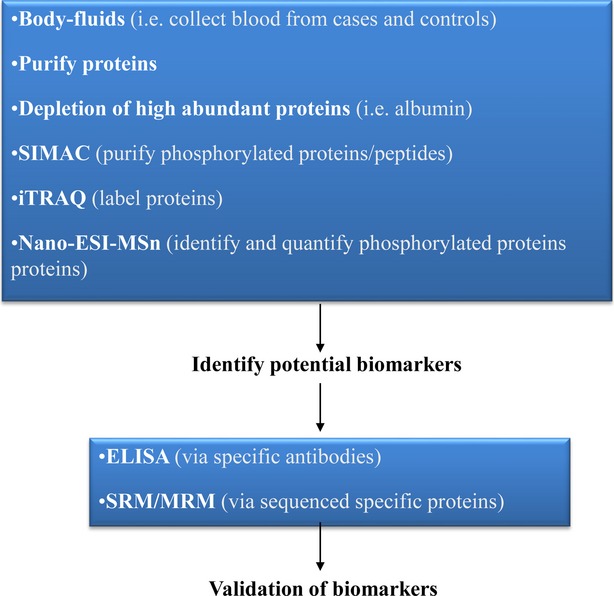
Possible flow-through – proteomic strategies – which may be useful for ALL-relapse research. The proteins must be purified from body-human fluids, subsequently high abundant proteins must be depleted as they can artefact the biomarkers-identification of ALL-relapses. The resulting purified proteins can be loaded into SIMAC chromatography to purify phosphorylated proteins. Finally, the peptides can be labelled *via* iTRAQ to quantify the level expression of phosphorylated proteins for a given clinical sample. The resulting potentially identified biomarkers by mass spectrometry, can be validated *via* ELISA and SRM/MRM. In addition, just by using iTRAQ plus mass spectrometry tools, the reference map of the proteome (ALL paediatric relapses) can be achieved studying the differential expressed proteins. Thus, new therapy targets can be discovered to improve current therapies for paediatric ALL relapses.

**Fig. 2:**
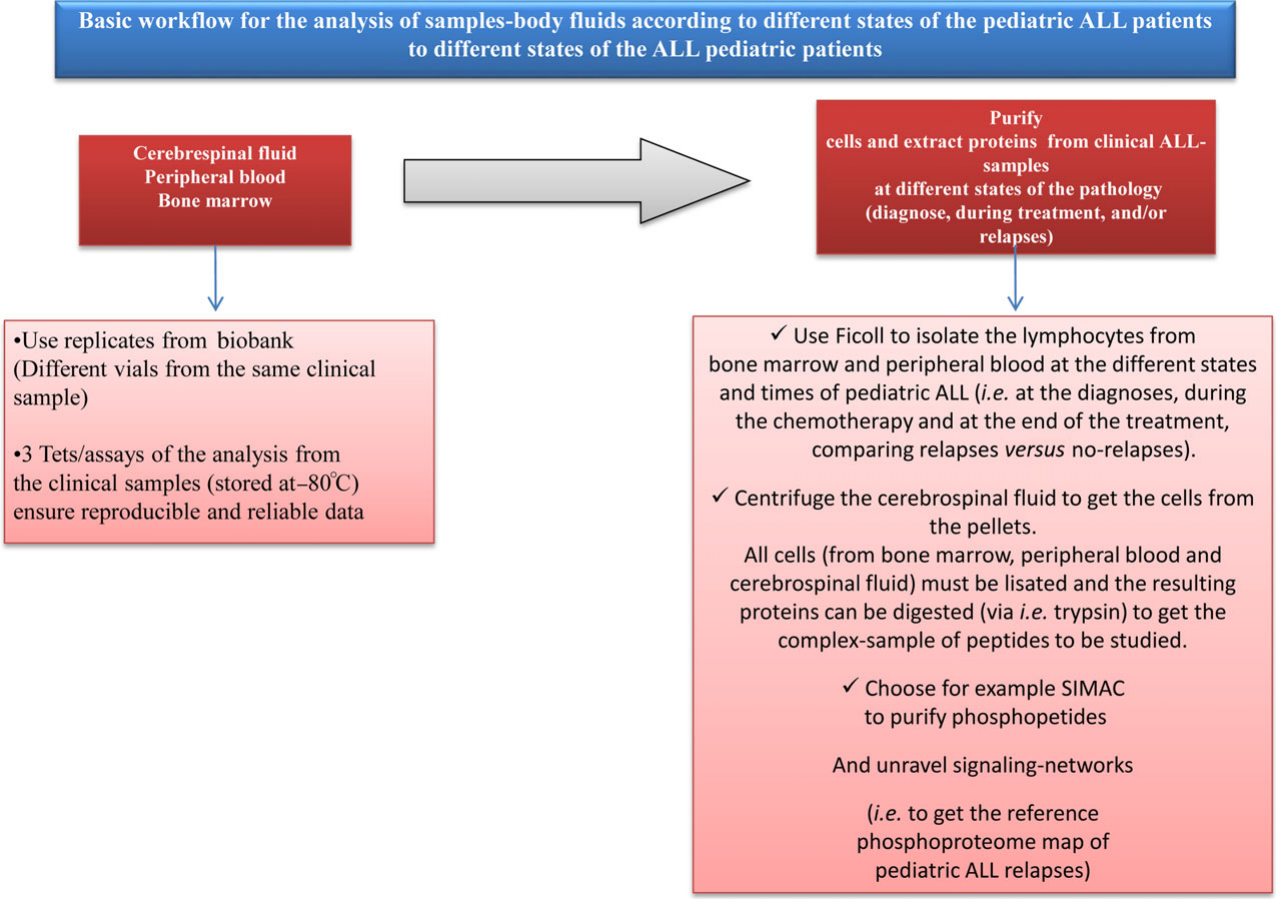
: Schedule for the analysis of sample-body fluids according to the different states of the paediatric ALL patients suffering relapses. The body-fluid samples, such as cerebrospinal fluid, peripheral blood and bone marrow from ALL paediatric patients at different states -at the diagnose, during chemotherapy and at the end of chemotherapy- must be analysed by using replicates (coming from different vials) to ensure reproducible data. First, the samples must be tested by using replicates from a biobank to get reproducibility and ensure the best conditions during all the work-flow, as the sample preparation is a critical step for achieving efficient data. Secondly, the lymphocytes from bone marrow and peripheral blood can be isolated *via* Ficoll, while the cells from the cerebrospinal fluid can be isolated by centrifugation. All cells from the three detailed kinds of samples must be lisated by using kits for human cells (*i.e*. RIPA) and the resulting proteins can be digested *via* trypsin to get the complex-mixture of peptides from the clinical sample. Finally, the resulting peptides can be loaded into SIMAC to isolate the phosphorylated peptides. Subsequently the purified phosphopeptides can be desalted, cleaned and concentrated (*i.e. via* POROS R3 reverse-phase chromatography) and injected in the mass spectrometer. The resulting data by mass spectrometry, permits improving the knowledge of signalling networks related to paediatric ALL relapses, which permits obtaining the phosphoproteome reference map of paediatric ALL-relapses, thus important clues of ALL-relapses can be unravelled. (Note: using 150 µg of proteins per sample (individual or *via* pools) is enough to carry out the complete proteomic strategy and identify biomarkers of paediatric ALL-relapses).

Flow-through –proteomic strategies may be useful for ALL-relapse research to innovate therapies *via* concepts (Figs. 1 and 2). The sample preparation of body fluids is an important and critical pre-requisite to achieve efficient data for proteomics [29]. For example, the cerebrospinal fluid (CSF) contains the low amount of proteins as compared to blood and can be even less at the end of the treatment, since leukemic cells are not present in CSF after the treatment. Conventional precipitations of acetone and TCA- acetone are used to isolate proteins from body fluids of patients with ALL. It is recommended that purified proteins of ALL complex mixtures be depleted of high abundant proteins (*i.e*. albumin). Efficient and reproducible data at expressions and PTMs of proteins can be achieved after the optimal isolation of proteins from cell lysis. Complementary data are obtained when the proteomic data from CSF and blood are analysed. Manual metal affinity chromatography is used to isolate multi-phosphopeptides *via* the immunoaffinity chromatography (IMAC) or mono-phosphopeptides *via* TiO_2_ from a biological complex-analyte. Metals with IMAC and TiO_2_ bring positive charge and link the negatively charged phosphopeptides [30]. The methodology of sequential elution of IMAC (SIMAC) is able to identify the phosphorylated residues from different types of human samples, samples between remission and relapse of ALL. Application of SIMAC can to increase the understanding of the activation of signalling pathways in the disease.

Isobaric Tag for Relative and Absolute Quantitation (iTRAQ) is more suitable for the simultaneous measurement of the relative and absolute quantification of 2–8 complex samples by a multiplexed isobaric chemical tagging reagent [31]. The advantages include the multiplexing of multiple complex-protein samples, production of identical mass spectrometry (MS)/MS sequencing ions for the derivatized tryptic peptide, and quantitation-based analyses to comparing between peak areas and peak ratios in MS/MS mode. It is more important to have a clear and practical strategy, including iTRAQ labelling for the three states of the pathology, determination of up-regulated and down-regulated proteins from ALL-relapse cases, and the expression level of phosphorylated proteins from the signalling pathways involved in ALL-relapses. About 15% of the total proteins between stable remission and relapse is considered as the standard of up or down-regulation, when the expression of protein is measured for clinical research. ElectroSpray Ionization and tandem Mass Spectrometry MS –*n* refers to tandem (nano-ESI-MSn) may represent one of the most sensitive, discriminating, and direct methods for the qualitative and quantitative high throughput analysis of sub-picomole amounts [32]. Nano-ESI-MS/MS is used to identify the labelled and isolated total and phosphor-proteins/peptides from complex samples and data analyses of the quantified and modified residues are conducted *via*, *i.e*. Matrix-Science Mascot searching (http://www.matrixscience.com/cgi/search_form.pl?FORMVER=2&SEARCH=MIS).

We identified candidates of protein biomarkers of ALL-relapses and explore the activation and interaction of signalling networks *via* the phosphorylation during ALL-relapses. The three dimensional strategy was considered, *e.g*. time and state after diagnosis, treatment, and remission of patients, and kind of samples, to draw a solid figure of selected proteins in the pathophysiological condition. The sample preparation for clinical proteomics should be paid the special attention because of clinical complexity [33]. The resulting identified protein biomarkers from ALL-relapses, can be validated by (a) ELISA and/or western blot [using specific antibodies (Abs) – Abs are chosen according to the identified biomarkers-] and (b) *via* SRM/MRM (Selected/Multiple reaction monitoring assays [34]. SRM or MRM is a method of MSn, which allows the selection of an ion with a particular mass in the first stage of a triple quadrupole. Then, fragmentation-reaction produces an ion-product coming from the selected precursor ion. Signal abundances of product-ions are indicative of the abundance of the peptides in the sample. It is, thus, possible to quantify the absolute copy numbers of a given protein (or a few number of proteins) of a cell from biological fluids. Thus, it allows identifying, quantifying and validating a specific putative protein-biomarker *via* (a) knowing the specific peptide/s sequences and (b) designing the proper mass spectrometry protocol for a specific putative analyte. It reaches the level of femtomole sensitivity.

The described issues would unravel the main clues between stable remission ALL cases and relapses. We propose this strategy to determine which proteins are up and down-regulated (and post-translationally modified) as a result of ALL-relapse. This would benefit diagnosis and improve therapy effectiveness for those patients. As many research groups (Hospitals, Universities, Research centres, Industry) could carry out this strategy and cooperate, it may be possible to collect all the information (according to different patients), thus the resulting data will be statistically higher and more refined (with obvious clinical advantages).

To summarize, the described developed proteomic tools imply advances for clinical research on paediatric ALL-relapses. We should point out that although 2DE-gels allowed stabilizing great research clinical studies, there is a drawback related to 2DE- electrophoresis: it chiefly allows to visualize high abundant proteins, thus, protein-biomarkers which are commonly low expressed can be lost *via* this tool. Nevertheless, 2DE is an interesting tool when carrying out isoform-proteins ALL studies. We consider that by using directly LC-MS/MS instead of –or avoiding- 2DE-gels, is an advantage, as more accurate and refine data can be achieved. In any case, the right proteomic strategy always depends on the goals of your research and the types of clinical samples to be studied. When performing with the detailed proteomic tools and following the work-flow (Figs. 1 and 2), real protein biomarkers of paediatric ALL-relapses can be discovered. This, undoubtedly, will contribute to innovate therapies for children with ALL suffering relapses.

### Examples of leukaemia research by using proteomics

Many leukaemia research studies should be mentioned. Here, we will show a few examples which we consider represent general and important proteomic flow-through according to the sample characteristics and the goals.

Hegedus *et al*. (2005) used surface-enhanced laser desorption/ionization time-off flight mass spectrometry (SELDI-TOF-MS) to analyse cell lysates from childhood leukaemia cell lines as well as pre-treatment leukemic bone marrow derived from childhood leukaemia cases [35,36]. Moreover, they carried out analyses of childhood leukaemia bone marrow comparing ALL and AML leukaemia types. Hegedus *et al*. (2005) observed differentially expressed proteins between AML and ALL-specific cell lines (ALL cell lines: Kasumi and the bi-phenotypic myelomonocytic cell line MV4;11; AML cell lines: 697 and REH) [35]. In this interesting research article, in particular, one 8.3 kDa protein was identified as a C-terminal truncated ubiquitin. In fact, it was noted that the role of this previously mentioned C-terminal truncated ubiquitin in the aetiology of ALL must be elucidated in the near future, as it could be a relevant clue for the diagnosis and therapy of ALL. The results of Hegedus *et al*. (2005) demonstrated the potential for proteome analyses to distinguish between various forms of childhood leukaemia in a simple manner [35].

Secondly, it is well known that chronic myeloid leukaemia (CML) is a disease guided *via* a molecular defect on the BCR-ABL translocation, resulting in: activation and dysregulation of a large number of signalling pathways, including cell division changes and also genetic-abnormalities incorporation [36–40]. In fact, BCR-ABL tyrosine kinase inhibitor was introduced in CML therapy as being one of the major advances in leukaemia treatments. Unfortunately, tyrosine kinase inhibitors are not effective for all patients. They fail in those patients who are subjected to blastic transformation, and are not able to extinguish CML at the stem cell level. In addition and regrettably, clinical drug-resistances can appear *via* the acquisition of BCR-ABL gene mutations [41]. To improve these issues, several new molecules were synthesized such as: IND-S1, MEL-T1, IND-S7 and MEL-S3 [42]. Turroni *et al*. (2013) synthesized new molecule-hybrids of spirocyclic ketones with antiproliferative, pro-apoptotic and differentiating activity in leukaemia cell lines, to unravel the action mechanisms of the previously synthesized molecules. In such an endeavour, the protein-expression profiles of K562 leukemic cells were treated with and without the compounds: IND-S1, MEL-T1, IND-S7 and MEL-S3, and subsequently, were analysed *via* two-dimensional gel electrophoresis (2DE) coupled to MS and bioinformatic tools by the authors [43]. The resulting proteome comparisons showed differentially expressed proteins related to: cellular metabolism, chaperone activity, cytoskeletal organization and RNA biogenesis. Turroni *et al*. (2013) validated the data observed *via* western blot and qPCR. In fact, they concluded that MEL-S3-treated leukaemia cells showed a marked expression of: glycoprotein IIb/IIIa (CD41) and glycoprotein Ib (CD42). Both glyco-proteins are two important protein-cell markers in megakaryocytic differentiation, together with morphological aspects of megakaryoblasts and megakaryocytes. Moreover, the megakaryocyte differentiation effect induced by MEL-S3 which was observed by Turroni *et al*. (2013), is correlated with the capacity of MEL-S3 to up-regulate EGR1 and HNF4 alpha transcript levels. The authors note that future research studies will confirm MEL-S3 improvements to CML conduct, potential therapeutic utilities in other malignancies with similar pathway alterations.

Another example we would like to mention is related to ALL and relapses. It is well known that during the treatment of acute leukaemia, drug resistance plays an important role in the patients' cure. Hu *et al*. (2011) applied proteomics tools *via* DIGE coupled to mass spectrometry (MS) by using a MALDI-TOF, to study the proteins whose expression level was affected *via* comparing between leukaemia cell line HL-60 and adriamycin-resistant HL-60 (HL-60/ADR) [44]. Hu *et al*. (2011) collected the clinical data from 80 patients. Moreover, the leukaemia cells from bone marrow and peripheral blood samples were taken before Chemotherapy treatment: 80 patients with newly diagnosed acute leukaemia, 16 patients in the setting of refractory disease, and 17 patients in relapsed disease, with an average age of 40. Hu *et al*. (2011) were able to identify 18 differentially expressed protein spots between the cell-lines: HL-60 and HL- 60/ADR *via* the previously mentioned proteomic strategy. From the 18 differentially expressed proteins, two were selected, according to their biological functions, for validation. These two proteins (Nucleophosmin/B23 (NPM B23) and nucleolin C23 (C23)) were validated *via* western blotting as they were up-regulated in leukaemia cells. Hu *et al*. (2011) according to their research studies concluded that Nucleophosmin/B23 (NPM B23) and nucleolin C23 (C23) are proteins directly involved in drug resistance issues [44].

Childhood ALL *versus* proteomics example Jiang *et al*. (2011) identified prognostic protein biomarkers in acute lymphoblastic childhood leukaemia (ALL) [45]. These authors were able to identify potential prognostic biomarkers and promising regulators of prednisolone-drug *via* proteomic strategies (2DE coupled to MS) and specific leukaemia cells of REH 697, Sup-B15 and RS4 11. Jiang N *et al*. (2011) used the drug prednisolone as it is well known that it implies a critical prognostic factor. This factor -consisting of early response to the treatment (prednisolone) during 1 week-, implies important prognostic factors in predicting eventual outcome in ALL. In this study, the selected mass spectrometer according to the proteomic strategy was MALDI-TOF/TOF. In MS mode, Jiang *et al*. (2011) identified 77 proteins, of which 17 showed modified protein-level expression when comparing prednisolone cell lines sensitive *versus* resistant [45]. Some of the proteins identified in this research study are: cell nuclear antigen (PCNA), cofilin 1, voltage-dependent anion-channel protein 1 (VDAC1) and proteasome activator subunit 2 (PA28β). In fact, these previously mentioned proteins were validated *via* western-blotting methodology, and the authors note that PCNA is a promising protein because of its important roles described in the literature, related to the cell cycle regulation and the cell-survival control.

During 2009, Shi *et al*. (2009) compared 94 paediatric ALL patients with 84 healthy controls [46]. This is one of the main paediatric ALL research clinical proteomic studies with more statistical significance with population from China. Linan *et al*. (2009) measured the proteomic serum profiles *via* SELDI-TOF-MS. Subsequently, the selected candidates were submitted to LC-MS/MS and validated *via* Protein Chip immunoassays. They identified as potential protein biomarkers of paediatric ALL: platelet factor (PF4), connective tissue activating peptide III (CTAP-III) and two fragments of C3a, to differentiate paediatric ALL patients from healthy controls and paediatric AML patients. Shi *et al*. (2009) remarked on the importance of using these three protein biomarkers to distinguish paediatric ALL patients from healthy controls and paediatric AML patients when analysing the protein profile of the serum [46]. It must be pointed out that these analyses were conducted with sensitivity and specificity values of ∼92% and ∼90%, respectively, meaning that future tests including additional populations would confirm the relevance of these diagnostic markers.

Casado *et al*. (2013) carried out phosphoproteomics to classify haematological cancer cell lines according to the kind of tumour and according to the sensitivity to kinase inhibitors [47]. Casado *et al*. (2013) *via* phosphoproteomics identified and quantified around 2000 phosphorylated residues in acute myeloid leukaemia (AML), lymphoma (LPH) and multiple myeloma (ML) cell lines. Interestingly, their data revealed that cell lines within a given disease (AML, LPH and MM) can be distinguished based on their specific phosphoprotein content. Indeed, they identified 601 up-regulated phosphorylated proteins in AML, LPH and MM, from which 544 are up-regulated specifically in AML, 30 in LPH and 27 in MM. Also, 107 phosphorylated proteins were down-regulated, from which 65 are specifically down-regulated in AML, 16 in LPH and 26 in MM. Casado *et al*. demonstrated that SU-DHL-6 and RL are phosphorylated proteins up-regulated while DoHH2 is a phosphorylated protein down-regulated specifically in LPH cell lines. MV4-11 and P31/Fuj are phosphorylated proteins up-regulated while CTS is a phosphorylated protein down-regulated specifically in AML cell lines, and RPMI-8226 and U26681 are phosphorylated proteins up-regulated while CMP2 CTS is a phosphorylated protein down-regulated specifically in MM cell lines [47].

In our opinion, this research work opens a new and important door to refine the current haematological therapies to reach the level of personalized medicine. They observed three main issues when applying phosphoproteomics for leukaemia research: the identification of phosphorylated residues from the proteins/peptides working in leukemic-cells that can be used to predict the response of the pharmaceutical drugs, the quantitative data of phosphorylation achieved *via* MS, manifest that the leukemic resistant cells can be distinguished according to their patterns of protein kinases activities. In addition, the identified activated kinases *via* phosphoproteomic analysis in leukemic cells, ‘are on the same wavelength’ as the phenotypic-responses of leukemic cells against the compounds which target kinase signallings. More interestingly, Casado *et al*. (2013) point out that when applying phosphoproteomics (*i.e*. IMAC and LC-MS/MS) coupled to quantitative proteomics strategies for leukaemia investigations, novel and real protein biomarkers –of resistance or sensitivity to drugs which target the signalling networks, will appear [47].

We would like to point out that Hjelle *et al*. (2010) detailed that studies on the myeloid leukemic (ML)-cell proteome will allow the identification of a large number of new biomarkers [48]. Indeed, they also indicate that the prediction of response to therapy -through identifying these markers- is an interesting avenue for future personalized medicine (several proteomic-MS strategies are explained for ML research studies in a magnificent way). Hjelle *et al*. (2010) presented a review showing several proteomic tools which obtained refined clinical data from ML cells and proteins *via*: SILAC, iTRAQ and DIGE, for biomarker discovery; and *via* RPPA (reverse-phase protein array: -RPPA is an antibody-based assay which detects and quantifies protein production-) and MRM, for biomarker validation. In addition, they distinguish the MRM tool as a highly sensitive method for ML clinical research, as it allows detection of slight differences in production of proteins identified as potential biomarkers. In addition, they explain that the flow cytometry methodology -being a characteristic and efficient tool for the analysis of leukemic cells- can be coupled to MS to solve the multiplexing drawbacks when detecting more than 20 protein biomarkers [48]. Refined molecular-therapeutic data allow monitoring therapy responses and Hjelle *et al*. (2010) explained in their review that protein analyses will play a supplementary and really prominent role in the future of molecular diagnostics of haematological diseases [48].

A recent ALL-research article (Braoudaki *et al.,* 2013) using 2DE-gels coupled to MALDI, shows that proteins such as CLUS, CERU, APOE, APOA4, APOA1, GELS, S10A9, AMBP, ACTB, CATA and AFAM are playing an important function in leukaemia prognosis, possibly acting as distinctive biomarkers for leukaemia aggressiveness, or as suppressor proteins in HR (high risk)-ALL paediatric cases [49].

The majority of these proteins were found to be up-regulated in HR and LR (low risk)-ALL bone-marrow (BM) and peripheral blood (PB) from paediatric patients at diagnosis when compared to non-leukemic patients (control). Nevertheless, GELS protein-expression was observed to be down-regulated in LR and HR-ALL patients when compared to non-leukemic patients. It is well known that GELS protein is involved in cytoskeletal changes during differentiation and carcinogenesis and is a potentially strong indicator of apoptosis. Furthermore, GELS has been suggested to play a prognostic role in various cancer types (*i.e*. breast cancer, brain tumour astrocytoma and childhood AML). Interestingly, in this research, BICR1 (Bicaudal D1) protein was shown to be up-regulated in all BM samples from LR-ALL paediatric patients compared to healthy ones. BICR1 is strong suppressor of PAR-driven (protease-activated-receptor-1), which plays a central role in cancer [49]. Indeed, BICR1 is associated with telomere-length variation in humans. Braoudaki *et al.,* (2013) propose that the identification of BICR1 in ALL paediatric-patients can be an early telomere dysfunction in these children. On the other hand, CLUS, CERU, APOE, APOA4, APOA1, GELS, S10A9, AMBP, ACTB, CATA and AFAM proteins are shown to be relevant regulators in the distinction between HR and LR-ALL *via* bioinformatic tools. Moreover, in this article, it is shown that vitronectin and plasminogen can be partly responsible for leukemogenesis, while bicaudal D-related protein 1 could possibly be a notable biomarker for paediatric ALL therapeutics [49].

Braoudaki *et al*. (2013) also observed that there is no direct linkage between specific protein signatures in LR and HR-ALL patients when correlating this data to cytogenetic abnormalities from each patient. Additionally, the resulting data were validated by western-blot analysis by using specific antibodies against the identified proteins and their protein-expression resulting data were analysed according to risk assessment and karyotype. The methodology followed by Braoudaki *et al.,* (2013) implies a very well designed strategy as they successfully coupled molecular biology techniques plus genetic analysis, proteomic assays and bioinformatic tools [49].

Finally, we aim to detail that Frank Stegmeier and coworkers (2013) carried out proteomic approaches to identify distinct molecular chromatin signatures profiling global histone modifications in human cancers [50]. They were able to identify specific chromatin finger-prints by mass spectrometry when comparing 115 cancer lines (from Cancer Cell Line Encyclopedia (CCLE): a collection of ∼1,000 human cancer lines that has undergone extensive genomic and pharmacologic annotation **[**[51]].

When they sequenced more than one thousand paediatric cancer genomes, NSD2p.E1099K alteration was detected in 14% of t(12;21) ETV6-RUNX1-containing acute lymphoblastic leukaemia. Indeed, several cancer lines bringing NSD2 translocations, showed an up-regulation of histone 3 lysine 36 (H3K36) dimethylation. Interestingly, an NSD2p.Glu1099Lys (p.E1099K) variant was also detected in non-translocated ALL cell lines which also contained the up-regulated H3K36. Additionally, *NSD2* mutations in the CCLE were hardly enriched in ALL lines, particularly the ones which come from paediatric ALL patients. To statistically validate *NSD2* mutations in primary paediatric ALL and see their prevalence in the different types of paediatric cancer, NSD2 coding exons were sequenced in approximately one thousand paediatric cancer samples. The result showed that the cohort of this study represents 21 different cancers and contained examples of all major subtypes of leukaemia, brain tumours and solid tumours seen in the paediatric population. Moreover, 18 ALL tumour and a low-grade glioma were found to harbour somatic mutations in *NSD2*, with the p.E1099K alteration being most prevalent (15/19 cases). On the other hand, *NSD2* mutations showed to be enriched in the B-ALL subtypes t(12;21) *ETV6-RUNX1* (20%) and t(1;19) *TCF3-PBX1* (15%) [50].

More generally, this brilliant approach offers a strategy to routinely profile chromatin states in many cancers to improve therapies. To summarize, Frank *et al*. (2013) applied global chromatin analyses *via* MS and detected and quantified the levels of histone modifications in bulk chromatin to visualize histone alterations related to cancer. They chose proteomics coupled to MS so as to remove the limitations concerning other methodologies (*i.e*. antibody-based methods, as not all the antibodies are currently available), but also because of the power of proteomics and other omics tools which are available thanks to efforts and innovations in these technologies during the last decade, thus providing us with the opportunity to use them for better more accurate diagnoses, and if possible better therapies [50,51].

## Conclusions and future perspectives

Nowadays, the scientific community is observing important research studies of leukaemia *via* proteomic strategies. In general, proteomic investigations allow the identification of a high number of proteins involved in the pathology leukaemia progression. We (scientists and clinicians) –after reading so many important articles on leukaemia research by using proteomic tools – can get specific information, but we still have not reached the total real protein biomarkers of ALL-paediatric relapses, which are so important to improve therapies for those children resistant to current therapies.

In such an endeavour, we are carrying out a group-consensus (among several hospitals and research centres) to stimulate and commence new research studies to improve and homogenize the resulting data of paediatric ALL (relapses). We consider that, with the application of this strategy (or similar ones) by many groups, more statistically refined data will be achieved, thus improving the clinical uses.

Individualized medicine is a concept which has gained popularity during the last decade. To reach such an important step in paediatric ALL –relapses-, experts in leukaemia (clinicians) and proteomics (biotechnologists) must work together, to establish homogeneous protocols, thereby improving the current data and obtaining new information. This will certainly improve and compact the relevant information we have obtained in the past, and will ultimate the efficacy/therapies *via* the future resulting data, as undoubtedly too little prognostic knowledge is known at paediatric ALL-relapse.
